# The Question of Treating the Poor Who Can Not Pay the Physician

**Published:** 1897-08

**Authors:** 


### THE QUESTION OF TREATING THE POOR WHO CANNOT PAY THE PHYSICIAN.

A few weeks ago a physician in Minneapolis, Minn., refused to attend an urgent call to see a patient that attempted suicide unless he received his pay in advance. As usual popular indignation was aroused against the doctor, the daily *Times* of that city going so far as to call such a refusal "professional cussedness." While we think that the refusal to act in such a case was wrong, still we can not help believe that most of the "cussedness" belongs to the men and women who expressed their indignation at the affair. But for their "cussedness" such a thing could not have occurred. Had they, as taxpayers, made arrangements so that any doctor could demand from the city, say half his usual fee in all emergency cases, nothing of the kind could occur. Why should these people, that set themselves up as judges of the doctor, refuse to put their hands into their pockets and divide the fee by paying part of it themselves? Why ask the doctor to bear the whole of the burden and take the whole risk? Such cases not only demand time in which he might earn more money while waiting upon them, but in many instances they mean hours or days from his duty, acting as a witness in court. We venture to assert that the indignant ones would in their own line of business be less generous than the average medical man is in his, where a case of suffering presents itself. We should like to know whether the editor of Minneapolis *Times* puts free advertisements into his paper for penniless and hungry men who are seeking work. Or does he permit them to commit suicide, and then speak of the doctor who imitates his example as a case of "professional cussedness?" Is the manager of the *Times* a "cuss" if he refuses free advertising to the poor?—*American Medical and Surgical Bulletin.*

